# The rocky road to recognizing interventional radiology as a full clinical speciality

**DOI:** 10.1186/s42155-020-00202-6

**Published:** 2021-01-06

**Authors:** Mohamad Hamady, Ian McCafferty

**Affiliations:** 1 Department of Interventional Radiology, Imperial College-London, Praed Street, London, W2 1NY UK; 2 Consultant Interventional Radiologist, Queen Elizabeth Hospital, Birmingham, UK

Over the past decades, interventional radiology (IR) has made significant progress in technological applications, quality of care, training, expansion, and even recognition. The historical challenges IR faces have deepened in the past ten years or so, following the remarkable success of image-guided minimally invasive surgery.

Serious Intrusions into the field and vicious turf competition, lack of proper recognition, suboptimal recruitment path and workforce issues, lack of recognized clinical training, mismanaged relation with diagnostic radiology, growth in cost and complexities of interventions as well as moral duty to ensure best medical care and protection for patients are among the threats and challenges facing modern IR. The biggest responsibility is how to turn these threats and challenges into opportunities.

All interventional radiologists know what needs to be done, and extensive discussions have been occurring for a long time. There is little argument that clinical practice, profile, clear identity, and advancing science are some of the main pillars for the specialty’s long-term survival. Achievements have been made in some areas—at a slow pace, but one could argue that these achievements have been too little too late.

Given the scale of IR’s success in almost all aspects of medicine, the increasing clinical responsibilities of interventionalists, and the inability to cope with massive growth of services and procedures in the field, IR’s survival will be at risk if the current structure, mentality, and mainstream ideology are not adjusted. The only way to secure a viable future career in IR for the coming generations is to establish a clinical specialty, which has biological links to radiology. Otherwise, IR could slip into a low key service, catering only for lines, biopsies and drainages. Furthermore, the successful transatlantic experience of restructuring IR and lessons learned are worth studying in depth. Time matters, and life does not forgive those who do not seize opportunities at the right time.

There is overwhelming enthusiasm and understanding among interventional radiologists regarding the need to augment IR’s status. Members of the British Society of Interventional Radiology, who constitute almost one fifth of the Royal College of Radiologists, voted anonymously in November 2019 in favor of recognizing IR as an independent specialty. However, currently, no country in the EU views IR as an independent specialty.

A list of underlying causes and hurdles can be blamed for this extended delay in making radical changes to IR status. To confront opposing powers and overcome hurdles, several strategic and tactical goals should be adopted, using a range of political, scientific, organizational, and educational tools. One of the most important actions should be highlighting the monetary value that IR provides to patients and health-care systems (Rubin 2017). An example is day-case services. Interventional radiology is positioned in a unique place, where units can offer integrated streamlined care pathways, from clinical assessment through radiological workup, interventions, and discharging patients to longitudinal care.

One of the few consolations of COVID-19 was the outstanding performance of IR units in many parts of the world (Rostampour et al. 2020). The contribution to patient care, when several other surgical and medical specialties caved in, should be distinctly and regularly highlighted to management and health policy makers. The ambulatory services that IR can offer extend well beyond vascular interventions to include oncology, palliative care, and pain management (Buss 2017). The safety of day-case vascular interventions was clearly documented over a decade ago (Huang et al. 2008). The recent documentation of Get It Right First Time (GIRFT) on Radiology report in the UK recommended day-case facilities as a way forward to improve cost-effective, high-quality health care (Halliday et al. 2020).

Interventional radiologists would benefit from taking on leadership roles at local and national levels, as well as engaging in the administrative aspects of health-care management. Interventional radiology’s involvement in senior hospital management allows its voice to be heard at a decision-making level.

Attention should be given to recruitment. A direct recruitment path to IR from year one of training, as will be the case in the UK beginning in 2022, is a crucial step, not only to obtain the most suitable candidates but also to shape the training curriculum. The clinical competency of the IR curriculum must receive priority attention by national training bodies. Parallel to this line of shaping future generations, involvement in education and organized events for medical students is equally important to spread the word and bust myths about IR among young trainees, especially female doctors (Xu et al. 2020).

Needless to say, national societies and CIRSE should continue to support the hard work and innovation, advancements in technology, and the promotion of evidence-based practice. Comprehensive data collection that shows the value for money as well as the quality of care that IR offers to patients will be a decisive tool to win the hearts and minds of politicians and the public alike.

It is also crucial to win over the cautious minds of one’s diagnostic radiology colleagues, who have a tendency to fixate on today’s issues rather than look at future benefits that fully functioning IR could offer. The conversation should be approached with positive and persuasive thinking to demonstrate the enormous benefits of a full clinical IR specialty to all parties and explain the serious implications if no action is taken. There is a real chance that this partnership between diagnostic radiology and IR could be a wonderful opportunity for mutual growth and expansion of the medical fields.

It is more important than ever that IR communities and individuals take personal and organizational responsibility to move toward an established clinical specialty that centralizes patient care and ensures viable career prospects for trainees.

Image-guided minimally invasive surgery is the future of medicine—but the role and existence of IR, if it continues in the current format, is threatened by viciously competing specialties. Finally, “I’ve learned; That opportunity is never lost; someone will take the ones you miss.” (Andy Rooney).

## Data Availability

Not applicable.
